# Combined Detection of Urinary Micro Albumin, α1-microglobulin and N-acetyl-β-D-glucosaminidase in the Early Diagnosis of Diabetic Nephropathy

**DOI:** 10.12669/pjms.336.13168

**Published:** 2017

**Authors:** Xuejun Zhang, Hongyan Zhou, Yan Li

**Affiliations:** 1 Xuejun Zhang, Clinical Laboratory, Binzhou People’s Hospital, Shandong, 256610, China; 2 Hongyan Zhou Clinical Laboratory, Binzhou People’s Hospital, Shandong, 256610, China; 3 Yan Li, Department of Blood Transfusion, Binzhou People’s Hospital, Shandong, 256610, China

**Keywords:** Diabetic nephropathy, Early diagnosis, Micro albumin

## Abstract

**Objective:**

To analyze the values of combined detection of urinary micro albumin (mAlb), α1-microglobulin (α1-MG) and N-acetyl-β-D-glucosaminidase (NAG) in the early diagnosis of diabetic nephropathy (DN).

**Methods:**

Ninety-four patients with early DN who were admitted to the hospital between April 2015 and April 2016 were selected and set as a DN group. Moreover, seventy-six patients with diabetes who were admitted to the hospital in the same period were selected and set as a diabetes group, and sixty-four healthy people were selected as set as a control group. The urinary mAlb, α1-MG and NAG of the three groups were detected. Moreover, the patients were divided into a favorable blood glucose control group and a poor blood glucose control group according to the blood glucose control condition of the patients. The detection results of the three groups were compared and statistically analyzed.

**Results:**

The urinary mAlb, α1-MG and NAG levels of the DN group were significantly higher than those of the diabetes group and control group, and the differences had statistical significance (P<0.05). The detection indicator values of the favorable blood glucose control group were much lower than those of the poor blood glucose control group, and the difference was statistically significant (P<0.05). The positive rate of the combined detection of mAlb, α1-MG and NAG levels was 90.2%, which was much higher than that of single indicator (P<0.05).

**Conclusion:**

Combined detection of urinary mAlb, α1-MG and NAG is sensitive in diagnosing early renal damages in DN patients.

## INTRODUCTION

Diabetic nephropathy (DN) is the major complication of diabetic microangiopathy. Early renal damages are difficult to be discovered because there are no obvious clinical manifestations in the early stage and results of conventional test items such as urinary protein, urea nitrogen and creatinine are usually normal. But the kidney has manifested apparent pathological changes before the appearance of albuminuria. DN can be irreversible and even evolve to terminal stage once renal impairment and persistent proteinuria occur.^1^,^2^ Therefore, the early diagnosis and inhibiting or delaying the occurrence and development of nephropathy are the important subjects in the current clinical research.^3^ To enhance the early diagnostic accuracy of DN, detection of relevant indicators is usually used for assisting diagnosis. This study discussed the values of the combined detection of mAlb, α1-MG and NAG in the early diagnosis of DN by analyzing 94 DN patients, 76 diabetes patients and 64 healthy people from the Binzhou People’s Hospital, aiming to provide an evidence for the formulation of treatment scheme in clinics.

## METHODS

### General data

Ninety-four patients with early DN who were admitted to the Binzhou People’s Hospital between April 2015 and April 2016 were selected and set as a DN group. Moreover, 76 patients with diabetes who were admitted to the hospital in the same period were selected and set as a diabetes group. Patients in both groups were diagnosed following WHO diabetes diagnostic criteria.^4^ The patients in the DN group had negative urine protein and 20 ~ 200 mg/24 h urinary albumin excretion rate, suggesting the manifestations of early renal injury. None of them had allergy, connective tissue diseases and renal lesions induced by infection or tumor or complications such as ketoacidosis and heart failure. Those who previously took drugs and hormones which can induce albuminuria and renal toxic drugs were excluded.

In the DN group, there were 52 males and 42 females, with an average age of 50.1±5.7 years (35~73 years) and an average disease course of 7.3±2.2 years (3~12 years); in the diabetes group, there were 43 males and 33 females, with an average age of 51.3±6.1 years (36~74 years) and an average disease course of 7.1±2.0 years (3~11 years). Sixty-four people who went to the hospital to receive physical examination and obtained normal examination results were selected and set as a control group. In the control group, there were 35 males and 29 females, with an average age of 50.2±8.9 years (33~76 years). The differences of gender, age and disease course between the three groups had no statistical significance (P>0.05). This study has been reviewed and approved by the ethics committee of the hospital, and the patients have signed an informed consent.

### Specimen collection

10 mL of fresh morning urine was collected from each subject and then centrifuged at 3000 r/min at a radius of 12 cm for 10 minutes. Then the supernate was separated and preserved at -20 °C. Moreover, 3 mL of fasting venous blood was collected from each subject on the same day. Serum was separated and detected for haemoglobin A1c (HbAlc).

### Detection methods

DXC800 fully automatic biochemical analyzer (Beckman, USA) and reagents which were purchased from Roche Diagnostics GmbH Co., Ltd. in German and Beijing Leadman Biochemical Inc. in China. Urinary mAlb was detected using enzyme linked immunosorbent assay (ELISA). Urinary α1-MG was detected using radiation and scattering turbidimetry. Urinary NAG was determined using endpoint method. All the detection procedures strictly followed instructions. The mAlb level lower than 20 mg/L, α1-MG lower than 15 mg/L and NAG lower than 15.7 U/L were determined as normal. HbAlc was measured using thin-column method. Control of blood glucose was evaluated as favorable if HbAlc was lower than 7%. The procedures followed the instructions. The item quality was controlled and non-specific disturbance was excluded to ensure the accuracy of results.

### Statistical analysis

Data were analyzed using SPSS version 21.0. Measurement data were expressed as mean ± standard deviation and processed by t test. Enumeration data were expressed as % and processed by Chi-square test. Difference was considered as statistically significant if P<0.05.

## RESULTS

### Detection results of urinary mAlb, α1-MG and NAG levels

The urinary mAlb, α1-MG and NAG levels of patients in the DN group were significantly higher than those of the diabetes group and control, suggesting statistically significant differences (P<0.05; Table-I).

**Table-I:** Comparison of mAlb, α1-MG and NAG levels between the three groups.

*Group*	*n*	*mAlb(mg/L)*	*α1-MG(mg/L)*	*NAG(U/L)*
DN group	94	48.54±5.68*^#^	16.03±3.64*^#^	18.08±0.52*^#^
Diabetes group	76	9.72±5.03	7.67±2.21	10.23±1.08
Control group	64	5.69±4.93	3.43±2.75	9.41±0.77

* ***Note:*** indicated P<0.05 compared to the control group;

# indicated P<0.05 compared to the diabetes group.

### Correlation between blood glucose condition and various indicators

The detection of HbAlc demonstrated that, 97 patients had favorable control of blood glucose and 73 patients had poor control. The HbAlc, mAlb, α1-MG and NAG levels of the poor blood glucose control group were higher than those of the favorable control group, and the differences were statistically remarkable (Table-II).

**Table-II:** Comparison of levels of indicators between groups with different blood glucose control.

*Group*	*n*	*HbAlc(%)*	*mAlb(mg/L)*	*α1-MG(mg/L)*	*NAG(U/L)*
Favorable blood glucose control group	97	6.22±0.56*	10.14±5.30*	4.58±2.13*	11.09±1.02*
Poor blood glucose control group	73	14.07±1.73	45.11±9.22	18.14±3.37	20.14±2.68

* ***Note:*** indicated P<0.05 compared to the poor blood glucose control group.

### The positive rates of detection based on single indicators and multiple indicators in the DN group

The positive rate of detection based on urinary mAlb, α1-MG and NAG was higher than that based on single indicators, and the difference had statistical significance (P<0.05; Table-III).

**Table-III:** Comparison of positive rates of detection based on single indicators and multiple indicators in the DN group.

*Indicators*	*Number of positive cases(n)*	*Positive rate(%)*
mAlb	64	68.1
α1-MG	59	62.8
NAG	67	71.3
mAlb+α1-MG+NAG	85	90.4*

* ***Note:*** indicated P<0.05 compared to detection based on single indicator.

## DISCUSSION

It is reported that, the incidence of DN is 10% among patients who have suffered from diabetes for more than five years and 20%~30% among patients who have suffered for more than ten years.^5^ In early stage of DN, the reduced negative charge components on glomerular basement membrane and the increased diameter of filtration pores on glomerular filtration membrane increases the excretion volume of proteins with moderate molecular weight.^6^ In physiological state, mAlb which is a micro proteins with a molecular weight of 69 kD and negative charges are usually unable to pass through filtration barriers due to the effects of pore size barriers and charge barriers. Once the integrity of glomeruli is damaged, the filtration of micro proteins increases. The appearance of albuminuria and the increased level of albuminuria are associated with the extent of damage of glomeruli.^7^,^8^ Urinary mAlb in the early stage of DN is usually more sensitive to routine urine test and renal function examination. The level of urinary mAlb increases significantly even if glomeruli experience mild damages. The results of this study suggested that the output volume of urinary mAlb of the patients with early DN was significantly higher than that of the control group. To discover early renal damages, detection of urinary mAlb is regarded as a conventional test item for DN patients.

α1-MG is a kind of glycoprotein synthesized by the liver and lymphocyte. The α1-MG with a molecular weight of 3.3 kD can pass through glomeruli freely, and 99% of them is reabsorbed and metabolized by renal tubules. When the reabsorption function of renal tubules is failed, the output volume of α1-MG will increase.^9^,^10^ In the early stage of DN, renal tubular epithelial cells fail to act as barriers because its structural integrity is damaged due to ischemia, inflammation and toxic substances; as a result, the reabsorption function fails, which leads to the remarkable increase of excretion volume.^11^ The results of this study suggested that the excretion volume of urinary α1-MG of patients with early DN was much higher than that of healthy people and patients with diabetes, indicating renal tubules have been damaged in the early stage of DN. Therefore, α1-MG is considered as one of the early sensitive markers for DN.

NAG, a kind of hydrolase, is extensively distributed in organs. NAG with a molecular weight of 130000 is not easy to be filtered by glomeruli. But when kidney convoluted tubules are damaged, lysosome will release a large amount of NAG, leading to the obvious increase of NAG in urine.^12^,^13^ It has been pointed out that NAG can be regarded as a sensitive marker for renal tubular damage.^14^ In the early stage of DN, the increase of glomeruli filtration pressure, the reduction of negative charges on filtration membrane and changes of gaping holes induce the increased filtration of proteins, activation of lysosome and increase of urinary NAG.^15^,^16^ The activity of urinary NAG is abnormal even when the excretion rate of urine proteins is normal. Therefore, the detection of activity changes of urinary NAG of patients with diabetes is beneficial to the early diagnosis of DN.

In this study, the mAlb, α1-MG and NAG levels of patients in the favorable blood glucose control group and poor blood glucose control group were detected. The results demonstrated that the levels of the three indicators of the latter group were higher than those of the former group, suggesting mAlb, α1-MG and NAG can reflect the renal damages and blood glucose control conditions of patients with diabetes. The joint detection of the three indicators can improve the detection rate of early DN and help clinical doctors formulate scientific and reasonable intervention measures to effectively control blood glucose and promote the recovery of renal functions. Moreover, the mAlb, α1-MG and NAG of the DN patients were observed to explore the clinical values of detection of single indicator or joint detection of multiple indicators in the early diagnosis of DN. Table-III demonstrates that the positive rate of joint detection of the three indicators was 90.4%, which was significantly different with the positive rates of detection of single indicator. It suggests that joint detection of multiple indicators is superior to detection of single indicator.

## CONCLUSION

Urinary mAlb, α1-MG and NAG can be regarded as the sensitive indicators for early diagnosis of DN. Joint detection of the three indicators before the appearance of proteinuria in DN patients can significantly improve the positive rate of DN diagnosis and is beneficial to the early diagnosis of renal injury, and moreover the levels of the three indicators can be used to identify the severity and location of DN early and reflect treatment effect, suggesting certain clinical values to the occurrence, development and treatment efficacy of DN.

### Authors’ Contribution

**XJZ & HYZ:** Study design, data collection and analysis.

**HYZ & YL:** Manuscript preparation, drafting and revising.

**XJZ & YZ:** Review and final approval of manuscript.
